# Effect of the Seasonal Climatic Variations on the Flavonoid Accumulation in *Vitis vinifera* cvs. ‘Muscat Hamburg’ and ‘Victoria’ Grapes under the Double Cropping System

**DOI:** 10.3390/foods11010048

**Published:** 2021-12-25

**Authors:** Hao-Cheng Lu, Wei-Kai Chen, Yu Wang, Xian-Jin Bai, Guo Cheng, Chang-Qing Duan, Jun Wang, Fei He

**Affiliations:** 1 Center for Viticulture and Enology, College of Food Science and Nutritional Engineering, China Agricultural University, Beijing 100083, China; luhc@cau.edu.cn (H.-C.L.); phyllis4yt@cau.edu.cn (W.-K.C.); wangyu_0919@cau.edu.cn (Y.W.); duanchq@vip.sina.com (C.-Q.D.); jun_wang@cau.edu.cn (J.W.); 2 Key Laboratory of Viticulture and Enology, Ministry of Agriculture and Rural Affairs, Beijing 100083, China; 3 Grape and Wine Research Institute, Guangxi Academy of Agricultural Sciences, Nanning 530007, China; b5629@126.com (X.-J.B.); berry713@163.com (G.C.)

**Keywords:** double cropping system, table grapes, flavonoid metabolism, high temperature, transcriptomics

## Abstract

Under the double cropping system, berries usually showed significant quality variations in the summer and winter seasons. In the two-year/four-consecutive-season study, two table grapes of ‘Muscat Hamburg’ and ‘Victoria’ were investigated to determine the phenolic compounds in their berries. Different from those of the summer season, the berries in the winter season suffered no high-temperature stress since veraison to harvest in 2014 and 2015. The variations in the season temperatures led to a higher anthocyanin concentration in the winter season berries of ‘Muscat Hamburg’ grapes than that in the summer berries, while the summer season berries had higher proportions of acylated and methylated anthocyanins than those in the winter season berries. Similar to the anthocyanins, the winter season berries also had a higher flavonol concentration in both varieties. Transcriptome analysis showed that the upregulated genes involved in the flavonoid pathway in the winter season berries were agreed with the changes found in the metabolites. However, the influence of the growing seasons on the flavanols was not consistent in the two varieties, and the variations in *VviLARs* between the grapes of ‘Muscat Hamburg’ and ‘Victoria’ might be the cause. This research helped us better understand the double cropping system and how the climate factors affected the phenolic compounds in the double cropping system.

## 1. Introduction

In South China, the subtropical humid monsoon climate was usually considered suboptimal for grape cultivation because of the extremely high temperature and the concentrated rainfall in the growing season, especially in summer [1]. However, table grapes had a rapid development in South China in recent years, such as in Guangxi province. Cheng et al. [2] showed that the viticultural area of Guangxi had increased by three times, and the annual production value increased from 246 million to 2.6 billion yuan (CNY) since the beginning of this century. The rain shelter cultivation and double cropping system techniques contributed to the booming development. The double cropping system was a common technique in the subtropical regions [3,4]. In these regions, the excessive rainfall in the summer led to fungal diseases and increased the rot incidence, which negatively affected the grape quality [4,5]. After applying the double cropping system, berries ripened earlier than those with the traditional single cropping system, making it possible to avoid the intense rainfall and heatwave in the summer season. Furthermore, berries in the winter season usually had a better quality than those in the summer season [6,7].

Flavonoid compounds are critical secondary metabolites in grapes, including anthocyanins, flavonols, flavanols, etc. [8]. These compounds are synthesized through the phenylpropanoid-flavonoid pathway and share the common upstream steps through phenylalanine ammonia-lyase (PAL) to flavanone 3-hydroxylase (F3H) [1]. The accumulation of flavonoids is sensitive to environmental changes such as temperature and light [9]. Previous studies showed that the relatively low temperature was beneficial for the accumulation of anthocyanins, while high temperature inhibited the synthesis of anthocyanins [10,11]. Cohen et al. [12] showed that diurnal temperature variation altered the initial rates of proanthocyanidin accumulation, which was correlated strongly with the expression of the core genes in the flavonoid pathway. As for the light effect, the increased cluster exposure could promote the synthesis of anthocyanins and flavonols, while shading treatment would inhibit the related metabolism of these compounds [13,14]. In addition, changes in the climate factors could also affect the portion of various flavonoids. For example, the high temperature would increase the proportion of coumalylated anthocyanins in the ‘Merlot’ grape [15]. Water deficit could increase the proportion of the 3′5′-hydroxylated and the methoxylated anthocyanins in the ‘Cabernet Sauvignon’ grape [16].

In the present study, we chose two table grapes that occupy a more extensive market than the wine grapes in South China to determine the variations in the flavonoid profiles. The ‘Victoria’ is a table grape variety cultivated in Puglia region at the Horticulture Research Institute of Bucharest [17]. The ‘Muscat Hamburg’ is a classical cultivar of black table grape grown in many parts of Europe, highly appreciated for its beautiful bunches and pleasant Muscat flavor [18]. Although many studies focus on the aroma characteristics of the two varieties, their phenolic profiles under the double cropping were few reported. There were significant climate variations between the summer and winter seasons under the double cropping system, which usually exceeded the effects of the vintages in the traditional viticultural regions. The results could help the researchers better understand how climate parameters affected grape phenolic compounds under the double cropping system, which might improve the understanding of the grape berries in response to the climate changes accompanied by extreme weather conditions in the future

## 2. Materials and Methods

### 2.1. Vineyard and Double Cropping System

Field experiments were performed in *V. vinifera* L. cvs. ‘Muscat Hamburg’ (MH) and ‘Victoria’ (V) grapevines (both grafted on ‘SO4′) in 2014 and 2015 in four consecutive growing seasons. Experiment site located in Guangxi Academy of Agricultural Sciences which was in South China (22°36′ N-108°14′ E, elevation 104 m). Vines were planted in 2007 and trained to a Y-shaped training system with 2 × 4/5 shoots per meter and 1.0 m cordon above ground. Rain-shelters were applied to all vines to prevent over-rainfall damage. The vineyard was north-south row orientation with inter- and intra-row spacing of 3.5 m × 1 m. The double cropping system in the experiment site was described by Chen et al. [1]. In each vintage, vines had two growing seasons: the summer season and the winter season. The summer season began in March and the grapes were harvested around late July and early August. Then vines were pruned and enforced with 2.5–3.0% hydrogen cyanamide to start the winter season. Vines were budburst in August and grapes were harvested in January next year.

### 2.2. Berry Sampling and Meteorological Data Collection

In each growing season, berries were sampled at four E-L stages [19]: (1) pea-size (E-L 31), (2) onset of veraison (E-L 35), (3) veraison complement (E-L 36) and (4) harvest (E-L 38). There were three biological replicates for each variety. For each replicate, 300 berries were randomly sampled from about 50 vines. Berries were immediately frozen in liquid nitrogen and stored at −80 °C for metabolite and transcriptome analysis.

Meteorological data was acquired from a local climate monitoring station within one kilometer away from the experiment site. Photosynthetically active radiation and temperature were recorded per hour. Accumulated rainfall was recorded per day.

### 2.3. Extraction of Grapes Phenolic Compounds

For each replicate, 100 berries were selected randomly to peel off the skins in frozen status. Skins were quickly frozen in liquid nitrogen and then grounded to powder. The powder was dried at −40 °C under vacuum conditions for 24 h.

The extraction of anthocyanins and flavonols in skins was according to a previous study [20]. Briefly, 0.1000 g (±0.0002 g) dried powder was accurately weighed and put into the centrifuge tube. Then the dried powder was macerated and sonicated in 50% (*v*/*v*) methanol in water (1.0 mL) for 20 min under a low temperature (4 °C) and dark condition. The mixture was centrifugated for 10 min at 8000 rpm to acquire the supernatant. The residues were re-extracted again and all the supernatants were combined.

The extraction of flavanols in skins was according to Liang et al. [21]. Briefly, 0.1000 g (±0.0002 g) dried powder was accurately weighed and put into the centrifuge tube. For the various flavan-3-ol units, 1 mL of phloroglucinol buffer (0.5% ascorbate, 300 mmol/L HCl and 50 g/L phloroglucinol in methanol) was used to mix the dried powder and incubated at 50 °C for 20 min. Then the mixture was neutralized with 1.0 mL aqueous sodium acetate (200 mM) and centrifuged for 15 min at 8000 rpm. The residue was extracted twice and all the supernatants were collected and combined. For the free flavan-3-ol monomers, 1 mL of 70% acetone with 0.5% ascorbate was used to mix the dried powder and then centrifuged for 15 min at 8000 rpm. The extraction of the residue was conducted twice and all the supernatants were collected and combined. Then the supernatants were dried using a nitrogen stream at 30 °C. The dried samples were dissolved in 200 μL acidified methanol with 1% (*v*/*v*) HCl and then neutralized with 200 μL aqueous sodium acetate (200 mM).

### 2.4. HPLC-MS Analysis of Phenolic Compounds in Grapes

Anthocyanins were analyzed with an Agilent 1100 series HPLC-MSD trap VL equipped with a diode array detector (DAD) and a Kromasil C_18_ column (250 × 4.6 mm, 5 μm). Mobile phase A was 2% formic acid in water and B was 2% formic acid in acetonitrile. The detailed LC procedures and MS conditions have been described by He et al. [22]. Anthocyanins were quantified using the malvidin-3-*O*-glucoside as the external standards and expressed as mg/kg berry fresh weight (FW). Flavonols were analyzed on an Agilent 1200 series HPLC-MSD trap VL equipped with a variable wavelength detector (VWD) and a Zorbax EclipseXDB-C_18_ column (250 × 4.6 mm, 5 μm). Mobile phase A consisted of acetonitrile/formic acid/water (50:85:865, *v*/*v*/*v*), and mobile phase B consisted of acetonitrile/methanol/formic acid/water (250:450:85:215, *v*/*v*/*v*/*v*). The detailed LC procedures and MS conditions were described by Chen et al. [1]. Flavonols were quantified using the quercetin-3-*O*-glucoside as the external standards and expressed as mg/kg berry fresh weight (FW). Flavanols were analyzed on an Agilent 1200 series HPLC system equipped with a Poroshell 120 EC-C18 column (150 × 2.1 mm, 2.7 μm) and Agilent 6410 QqQ instrument equipped with an electrospray ionization source. Mobile phase A was aqueous 0.1% formic acid and B was a mixture of acetonitrile/methanol (50:50, *v*/*v*) containing 0.1% formic acid. The detailed LC procedures and MS conditions have been described by Chen et al. [1]. Flavanols were quantified by using (+)–catechin, (–)–epicatechin, (–)–epicatechin-3-*O*-gallate and (–)–epigallocatechin as the external standards and expressed as mg/kg berry fresh weight (FW).

### 2.5. RNA Extraction and Transcriptome Sequencing

Berries from three sampling points were selected (E-L 35, 36, and 38) for the RNA extraction. In this case, 50 berries were randomly selected from each biological replicate. Then the berries were de-seeded and smashed into powder under liquid nitrogen protection. The SpectrumTM Plant Total RNA Kit (Sigma-Aldrich, Carlsbad, CA, USA) was used for the total RNA extraction. Transcriptome analysis was conducted on the Illumina HiSeqTM 2000 platform with 50-bp single reads and aligned against the reference grapevine genome 12 × V2, allowing no more than two mismatches. The longest transcript was chosen to calculate the fragments per kilobases per million reads (FPKM) value when more than one transcript was obtained for a single gene. The R package ‘DESeq2′ was used to identify differentially expressed genes (DEGs), and the criteria were set as false discovery rate ≤ 0.05 and fold change ≥ 2. The data have been deposited in the NCBI Gene Expression Omnibus (GEO) database and are accessible through GEO accession GSE168785.

### 2.6. Statistical Analysis

The SPSS version 22.0 was used for all significance analysis at *p* < 0.05 (Duncan’s multiple range test or *t*-test). The figures were prepared by using GraphPad Prism 8.0.2 (GraphPad Software, San Diego, CA, USA) and R statistical environment (3.6.1). Heatmap was prepared using the ‘pheatmap’ package in R. Principal component analysis (PCA) and orthogonal partial least-squares discrimination analysis (OPLS-DA) were performed in SIMCA 14.1 (Umetrics, Umea, Sweden).

## 3. Results

### 3.1. Meteorological Data

The climate conditions of each development stage in 2014 and 2015 were shown in Table 1. Stage I was from the full bloom to the veraison beginning. Stage II was from the veraison beginning to the veraison completement. Stage III was from the veraison completement to the harvest. The ‘Muscat Hamburg’ and ‘Victoria’ grapes had similar phenological stages in the winter seasons of 2014 and 2015 (Supplementary Table S1). So they experienced similar climate conditions in these seasons. In the summer season of 2015, the ‘Muscat Hamburg’ grapes were harvested 23 days later than the ‘Victoria’ grapes. In the summer season, the GDD was significantly higher than in the winter season. The average daily temperature in the summer season was about 30 °C in three development stages. While in the winter season, only stage I could be up to 23 °C in terms of average daily temperature. In stage III of the winter season, the average temperature was no more than 16 °C, which indicated a cool weather station during the ripening period. Notably, the high-temperature hours in the summer season were at least three folds higher than those in the winter season. Furthermore, during stage II and stage III, almost no high-temperature weather occurred in the winter season. For the cumulative PAR/sunshine hours and the rainfall, there were no consistent trends in 2014 and 2015. In 2014, the winter season had more cumulative PAR/sunshine hours and rainfall than the summer season. However, in 2015, the winter season had fewer cumulative PAR/sunshine hours and rainfall than the summer season.

### 3.2. Anthocyanin Composition

In the winter season, the anthocyanin concentration of the berries was at least seven-fold higher than that in the summer season, as shown in Table 2. For individual compounds, 5 monoglycoside, 3 acetylated and 4 coumarylated anthocyanins were detected by HPLC-MS. Most anthocyanin compounds showed higher concentrations in the winter season than in the summer season, except for malvidin-3-*O*-acetylglucoside. In the two-way ANOVA, only the concentration difference of malvidin-3-*O*-acetylglucoside did not reach a significant level (*p* < 0.05) under the effect of the season. Only delphinidin-3-*O*-acetylglucoside, petunidin-3-*O*-coumarylglucoside and peonidin-3-*O*-coumarylglucoside were not affected by the vintage. Only malvidin-3-*O*-glucoside, peonidin-3-*O*-acetylglucoside and malvidin-3-*O*-acetylglucoside were not affected by the interaction of the season and the vintage. Peonidin-based anthocyanins occupied the highest proportion of all anthocyanin groups in ‘Muscat Hamburg’ grapes, ranging from 44% to 60%. The winter season berries significantly increased the proportion of cyanidin-based anthocyanins, while decreasing the proportion of malvidin-based anthocyanins.

**Figure 1:**
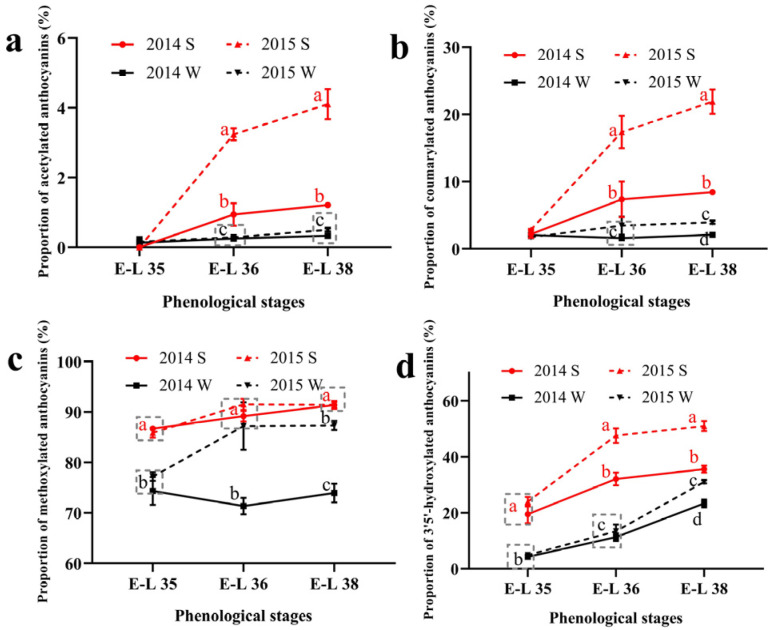
The proportion of acylated (**a**), coumarylated (**b**), methylated (**c**), and 3′5′-hydroxylated (**d**) anthocyanins in Muscat Hamburg grape in 2014 and 2015 under the double cropping system. Different letters within the same development stage indicate significant differences among seasons (Duncan’s multiple range test at *p* < 0.05).

The proportion of the acylated and the B-ring substituted anthocyanins was calculated and shown in Figure 1. For acylated anthocyanins, the summer season berries had higher proportions of the acetylated and the coumarylated anthocyanins than the winter season berries in both of the two vintages. The proportion of the acetylated and the coumarylated anthocyanins increased consistently during the growing season in the summer season berries while keeping stable in the winter season berries. As for the methylated anthocyanins, the summer season berries also had a higher proportion than the winter season berries. The increased proportion of malvidin-based anthocyanins and decreased proportion of cyanidin-based anthocyanins caused a higher proportion of the methylated anthocyanins in the summer season berries. In terms of the 3′5′-hydroxylated anthocyanins, their proportions followed an increasing trend in all the growing seasons. Similar to acylated and methylated anthocyanins, the proportion of 3′5′-hydroxylated anthocyanins was also higher in the summer season berries than in the winter season berries. The 3′5′-hydroxylated anthocyanins consist of the malvidin-based, the delphinidin-based, and the petunidin-based anthocyanins. Among the three types of anthocyanins, the increased proportion of the malvidin-based anthocyanins caused a higher proportion of the 3′5′-hydroxylated in the summer season berries.

### 3.3. Flavonol Composition

**Figure 2:**
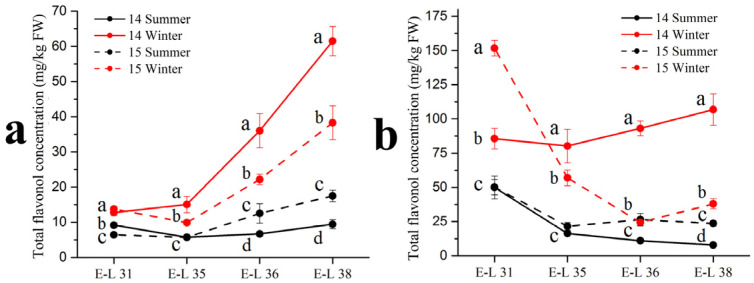
Total flavonol concentration in Muscat Hamburg grape (**a**) and Victoria grape (**b**) in 2014 and 2015 under the double cropping system. Different letters within the same development stage indicate significant differences among seasons (Duncan’s multiple range test at *p* < 0.05).

As shown in Table 3, 13 individual flavonol compounds were detected by the HPLC-MS, with 12 flavonols identified in the ‘Muscat Hamburg’ grapes and 8 flavonols identified in the ‘Victoria’ grapes. Among all the compounds, only quercetin-3-*O*-rutinoside was not detected in the ‘Muscat Hamburg’ grapes. Among all six types of flavonols, only the myricetin based flavonols had a higher proportion in the winter season berries than in the summer season berries in the ‘Muscat Hamburg’ grapes. The 3′5′-hydroxylated flavonols, such as myricetin-3-*O*-glucuronide, myricetin-3-*O*-galactoside, myricetin-3-*O*-glucoside, laricitrin-3-*O*-glucoside and syringetin-3-*O*-glucoside, were not detected in the ‘Victoria’ grapes. Mattivi et al. [23] showed that the delphinidin-like flavonols myricetin, laricitrin and syringetin were missing in all the white varieties, indicating that the enzyme flavonoid 3′,5′-hydroxylase was not expressed in the white grape varieties. Similar to anthocyanins, the summer season berries had lower concentrations of most flavonol compounds, leading to a significant reduction in total flavonol concentration (Figure 2). At harvest (E-L 38), the same result occurred in the two varieties in total flavonol concentration among different seasons. The 2014 winter season berries had the highest flavonol concentration among all the four seasons, followed by the 2015 winter season berries and the 2015 summer season berries. The 2014 summer season berries had the lowest flavonol concentration among all the four seasons in the two varieties.

### 3.4. Flavanol Composition

**Figure 3:**
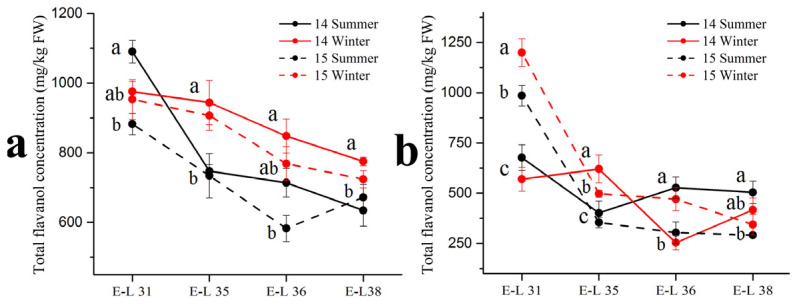
Total flavanol concentration in ‘Muscat Hamburg’ grapes (**a**) and ‘Victoria’ grapes (**b**) in 2014 and 2015 under the double cropping system. Different letters within the same development stage indicate significant differences among seasons (Duncan’s multiple range test at *p* < 0.05).

Respect to flavonols in the grape skins, the basic monomer (+)–catechin, (–)–epicatechin, (–)–epicatechin-3-*O*-gallate and (–)–epigallocatechin were detected by the HPLC-MS in three forms: terminal subunits, extension subunits and free monomers (Table 4). Among the four basic monomers, (–)–epicatechin occupied the highest proportion (approximately 70%) of the total flavanol concentration. There were no consistent trends in the (–)–epicatechin concentration between the summer and winter season berries in the two varieties. In 2014, the winter season berries had a higher (–)–epicatechin concentration than the summer season berries in the ‘Muscat Hamburg’ grape, while had an opposite result in the ‘Victoria’ grapes. However, in 2015, there was no significant difference in (–)–epicatechin concentration between the summer and the winter season berries. For other compounds, only (–)–epicatechin-3-*O*-gallate showed significant differences between the summer and the winter season berries in the two varieties. The summer season berries had a higher (–)–epicatechin-3-*O*-gallate concentration than the winter season berries in 2014 but the opposite result showed in 2015. In terms of total flavanol concentrations in the summer and the winter season berries (Figure 3), they showed decreased trends along the development stages which peaked at E-L 31. In the ‘Muscat Hamburg’ grapes, the 2014 winter season berries had the highest flavanol concentration among all the four seasons at harvest. While in the ‘Victoria’ grapes, the 2014 summer season berries had the highest flavanol concentration among all the seasons at harvest.

### 3.5. Principal Component Analysis (PCA) and Orthogonal Partial Least-Squares Discrimination Analysis (OPLS-DA) Based on the Phenolic Profiles at Different Stages

**Figure 4:**
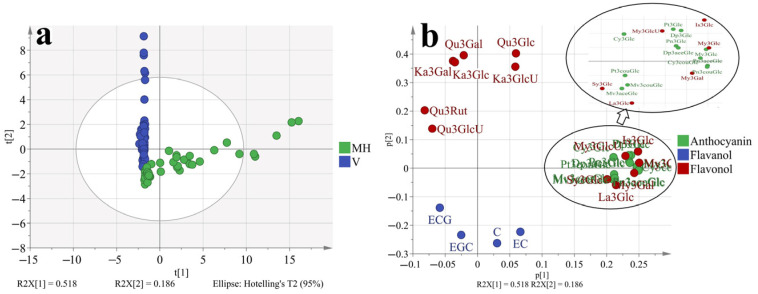
PCA (**a**, score plot; **b**, loading plot) based on the concentration of individual phenolic compounds in ‘Muscat Hamburg’ grapes and ‘Victoria’ grapes in 2014 and 2015 under the double cropping system. Dp3Glc, delphinidin-3-*O*-glucoside; Cy3Glc, cyanidin-3-*O*-glucoside; Pt3Glc, petunidin-3-*O*-glucoside; Pn3Glc, peonidin-3-*O*-glucoside; Mv3Glc, malvidin-3-*O*-glucoside, Dp3aceGlc, Delphinidin-3-*O*-acetyl-glucoside; Pn3aceGlc, peonidin-3-*O*-acetylglucoside; Mv3aceGlc, malvidin-3-*O*-acetyl-glucoside; Cy3couGlc, cyanidin-3-*O*-coumaryl-glucoside; Pt3couGlc, petunidin-3-*O*-coumaryl-glucoside; Pn3couGlc, peonidin-3-*O*-coumaryl-glucoside; Mv3couGlc, malvidin-3-*O*-coumarylglucoside; My3GlcU, myricetin-3-*O*-glucuronide; My3Gal, myricetin-3-*O*-galactoside; My3Glc, myricetin-3-*O*-glucoside; Qu3Gal, quercetin-3-*O*-galactoside; Qu3GlcU, quercetin-3-*O*-glucuronide; Qu3Rut, quercetin-3-*O*-rutinoside; Qu3Glc, quercetin-3-*O*-glucoside; La3Glc, laricitrin-3-*O*-glucoside; Ka3Gal, kaempferol-3-*O*-galactoside; Ka3GlcU, kaempferol-3-*O*-glucuronide; Ka3Glc, kaempferol-3-*O*-glucoside; Is3Glc, isorhamnetin-3-*O*-glucoside; Sy3Glc, syringetin-3-*O*-glucoside; ECG, epicatechin-3-*O*-gallate; EGC, epigallocatechin; C, catechin; EC, Epicatechin.

To better understand how the phenolic profiles varied in ‘Muscat Hamburg’ grapes and ‘Victoria’ grapes, as well as in different seasons. The principal component analysis (PCA) was used to classify the different samples, which consisted of all the four development stages of two varieties in different seasons, as shown in Figure 4 and Supplementary Figure S1. The first two principal components explained 70.4% of the total variance. PC1 accounted for 51.8% of the total variance, which could separate samples from MH and V grapes (Figure 1a). In the loading plot (Figure 1b), PC1 was characterized by all the anthocyanins and some of the flavonols. The V grapes had no anthocyanins so all the anthocyanins were located on the positive of axis x, which was in agreement with the previous analysis. Furthermore, only quercetin and kaempferol-based flavonols were detected in the V grapes. Other types of flavonols were also located on the positive of axis x. Except for the variations in different varieties, the samples from different seasons and development stages were also marked in the PCA, as shown in Supplementary Figure S1. However, the PCA could not separate these samples clearly. So the variations in the variety characteristics beyond the effects of growing seasons and development stages on grape phenolics. However, with respect to the summer and winter season effects (Supplementary Figure S1b), the samples from summer seasons were close to the coordinate origin, while some samples from winter seasons were abundant in anthocyanins and flavonols.

**Figure 5:**
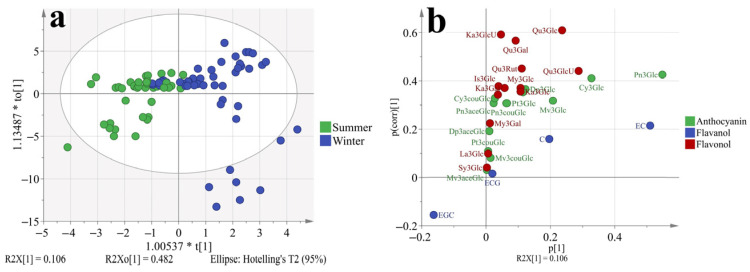
OPLS-DA (**a**, score plot; **b**, S-plot) based on the concentration of individual phenolic compounds in ‘Muscat Hamburg’ grapes and ‘Victoria’ grapes in the years of 2014 and 2015 under the double cropping system. Dp3Glc, delphinidin-3-*O*-glucoside; Cy3Glc, cyanidin-3-*O*-glucoside; Pt3Glc, petunidin-3-*O*-glucoside; Pn3Glc, peonidin-3-*O*-glucoside; Mv3Glc, malvidin-3-*O*-glucoside, Dp3aceGlc, Delphinidin-3-*O*-acetyl-glucoside; Pn3aceGlc, peonidin-3-*O*-acetylglucoside; Mv3aceGlc, malvidin-3-*O*-acetyl-glucoside; Cy3couGlc, cyanidin-3-*O*-coumaryl-glucoside; Pt3couGlc, petunidin-3-*O*-coumaryl-glucoside; Pn3couGlc, peonidin-3-*O*-coumaryl-glucoside; Mv3couGlc, malvidin-3-*O*-coumarylglucoside; My3GlcU, myricetin-3-*O*-glucuronide; My3Gal, myricetin-3-*O*-galactoside; My3Glc, myricetin-3-*O*-glucoside; Qu3Gal, quercetin-3-*O*-galactoside; Qu3GlcU, quercetin-3-*O*-glucuronide; Qu3Rut, quercetin-3-*O*-rutinoside; Qu3Glc, quercetin-3-*O*-glucoside; La3Glc, laricitrin-3-*O*-glucoside; Ka3Gal, kaempferol-3-*O*-galactoside; Ka3GlcU, kaempferol-3-*O*-glucuronide; Ka3Glc, kaempferol-3-*O*-glucoside; Is3Glc, isorhamnetin-3-*O*-glucoside; Sy3Glc, syringetin-3-*O*-glucoside; ECG, epicatechin-3-*O*-gallate; EGC, epigallocatechin; C, catechin; EC, Epicatechin.

To better discriminate the effect of different growing seasons on grapes phenolic profiles. The OPLS-DA model was used which had a better focus toward the studied objective than PCA, as shown in Figure 5. The model has passed 200 permutation tests (Supplementary Figure S2), indicating good fit and predictive abilities. Results showed that the samples from the summer and winter seasons could be separated better than in Supplementary Figure S1b. In the S-plot (Figure 5b), most phenolic compounds were located on the first quadrant of the coordinate axis except for EGC. So it was confirmed that the winter berries had more abundant phenolic compounds irrelevant with varieties or other factors. The peonidin-3-*O*-glucoside contributed the highest variation in all phenolic compounds. In the winter berries, the peonidin-3-*O*-glucoside was at least 8 folds higher than in summer berries, which was shown in Table 2. For epigallocatechin, there was no significant difference between summer and winter berries.

### 3.6. Flavonoid Biosynthesis

**Figure 6:**
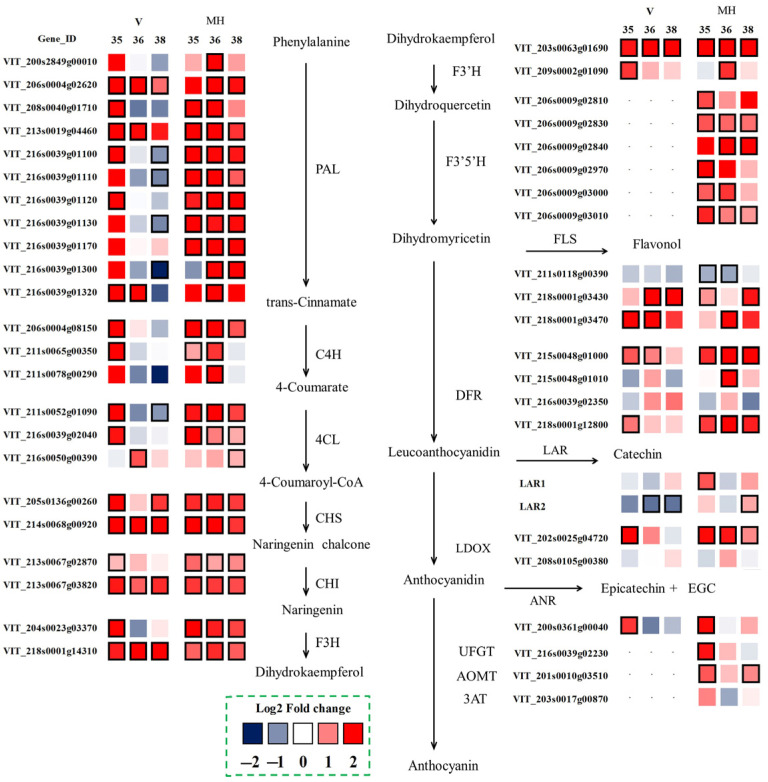
Effect of the growing season on the expression profiles of the flavonoid synthesis pathway during the development of the ‘Muscat Hamburg’ and ‘Victoria’ grapes in 2014. Heatmaps showed the log_2_ fold changes between seasons (winter season/summer season). Red block indicated higher gene expression in the winter season berries. Blue block indicated lower gene expression in the winter season berries. Boxes with bold margins indicated differentially expressed genes between the summer and the winter season berries.

To further understand the variation between the summer and the winter season berries at the transcriptome level, the differentially expressed genes related to flavonoid biosynthesis were selected, as shown in Figure 6. The biosynthesis of flavonoids shared the common upstream pathway through phenylalanine to dihydrokaempferol. Some important enzymes such as phenylalanine ammonia-lyase (PAL), cinnamate 4-hydroxylase (C4H), 4-coumarate: CoA ligase (4CL), chalcone synthase (CHS), chalcone isomerase (CHI), flavanone 3-hydroxylase (F3H) were all involved in this part [24]. In the ‘Muscat Hamburg’ grapes, almost all the genes had higher expressions in the winter season berries than in the summer season berries in the upstream pathway. Even at E-L 38, these genes still had higher expression in the winter season berries. In the ‘Victoria’ grapes, the selected differentially expressed genes had higher expressions at E-L 35 and/or at E-L 36, while downregulated at E-L 38, such as *VviPAL* (VIT_216s0039g01100) and *Vvi4CL* (VIT_211s0052g01090). Flavonoid 3′-hydroxylase (F3′H) and flavonoid 3′5′-hydroxylase (F3′5′H) were involved in the two branch pathways of catalyzing the synthesis of the 3′-substituted and the 3′5′-substituted flavonoids, respectively. There were two *VviF3′Hs* (VIT_203s0063g01690 and VIT_209s0002g01090) selected as the differentially expressed genes in the summer and the winter berries in the two varieties. Both of the two selected *VviF3′Hs* had higher expression in the winter season berries, especially VIT_203s0063g01690, which had a higher expression at E-L 35, E-L 36 and E-L 38. As for F3′5′H, six *VviF3′5′Hs* were differentially expressed in the summer and the winter season berries in the ‘Muscat Hamburg’ grapes, while the *VviF3′5′Hs* were almost not expressed in the ‘Victoria’ grapes (Supplementary Table S2). Different from the red grape varieties, the extremely low expressions of *VviF3′5′Hs* in the white grapes suggested that the enzyme flavonoid 3′,5′-hydroxylase was not expressed in their skins [23,25].

The downstream pathway of the flavonoid metabolism included multiple branches. The main related enzymes in these branches included flavonol synthase (FLS), dihydroflavonol reductase (DFR), leucoanthocyanidin dioxygenase (LDOX), leucoanthocyanidin reductase (LAR), anthocyanidin reductase (ANR) and UDP glucose: flavonoid 3-*O*-glycosyltransferase (UFGT). FLS was the key enzyme in the biosynthesis of flavonols. Among the five known *VviFLSs*, only *VviFLS4* (VIT_218s0001g03470) and *VviFLS5* (VIT_218s0001g03430) were reported to express in grapes [26]. In the present study, *VviFLS4* had a low expression in the ‘Muscat Hamburg’ grapes while it had a higher expression in the 2014 winter season of the ‘Victoria’ grapes (Supplementary Table S2). Compared to the summer season berries, the winter season berries had higher expressions of *VviFLS4* and *VviFLS5* than the summer season berries in both varieties. LAR and ANR were key enzymes in the production of the flavan-3-ol monomers required for the formation of proanthocyanidin polymers [27]. Two *VviLARs* and one *VviANR* were selected as the differentially expressed genes between the summer and the winter season berries. *VviLAR1* (VIT_201s0011g02960) and *VviLAR2* (VIT_217s0000g04150) were upregulated in the ‘Muscat Hamburg’ winter season berries. However, in the ‘Victoria’ grapes, *VviLAR2* was downregulated in the winter season berries. The genes involved in the synthesis and modification of anthocyanins have been widely studied, including *VviUFGT* (VIT_216s0039g02230), *VviAOMT* (VIT_201s0010g03510) and *Vvi3AT* (VIT_203s0017g00870) [28,29,30]. These genes were only expressed in ‘Muscat’ Hamburg grapes. The expressions of *VviUFGT* and *VviAOMT* were upregulated in the winter season berries, which was consistent with the coordinated expression of the upstream genes.

### 3.7. Expression Profiles of Flavonoid Related Transcription Factors

**Figure 7:**
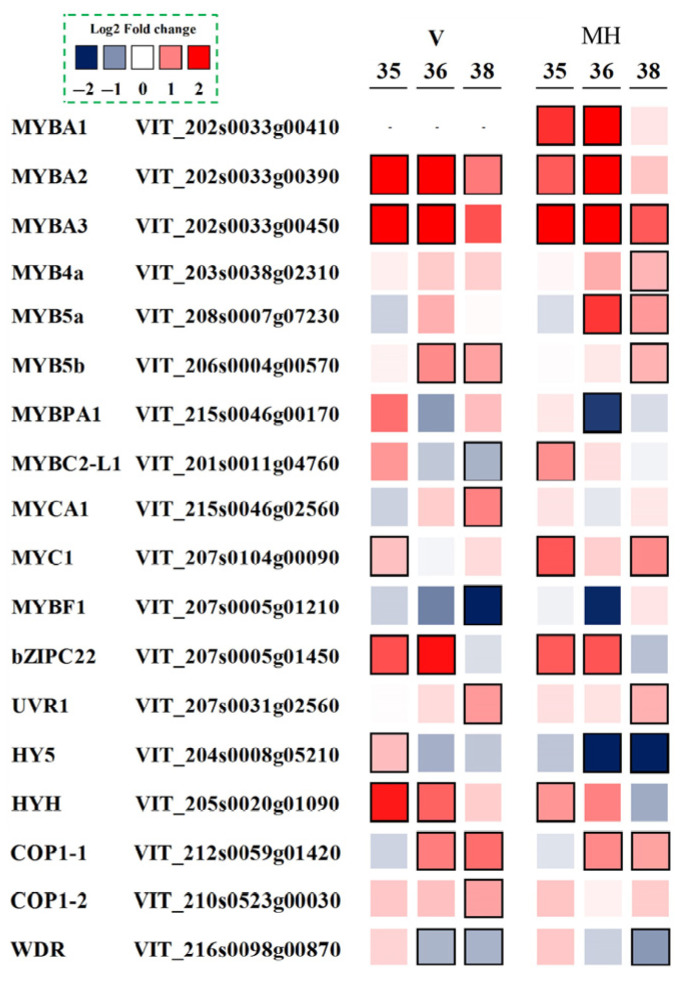
Effect of the growing season on the expression profiles of the flavonoid related transcription factors during the development of ‘Muscat Hamburg’ and ‘Victoria’ grapes in 2014. Heatmaps showed the log_2_ fold changes between seasons (winter season/summer season). Red block indicated the higher gene expression in the winter season berries. Blue block indicated the lower gene expression in the winter season berries. Boxes with bold margins indicated differentially expressed genes between the summer and the winter season berries.

In addition to the structural genes that encode enzymes in the flavonoid pathway, the related regulatory genes that control the transcription of these biosynthetic genes were also analyzed, as shown in Figure 7. Genes of the MYBA family were involved in regulating the anthocyanin biosynthesis in the grapes via regulating the expression of the UFGT gene [31,32]. *MYBA2* and *MYBA3* were expressed in both ‘Muscat Hamburg’ and ‘Victoria’ grapes and upregulated in the winter season berries, while *MYBA1* was only expressed in ‘Muscat Hamburg’ grapes and upregulated in the winter season berries. The regulators named *VviMYBPA1*, *VviMYB5a*, *VviMYB5b* and *VviMYBC2-L1* were involved in the regulation of the proanthocyanidin-specific biosynthesis [33,34,35,36]. In the winter season berries, the expressions of *VviMYB5a* and *VviMYB5b* were upregulated in both varieties, while *VviMYBPA1* was downregulated at E-L 36 in the ‘Muscat Hamburg’ grapes. Similarly, the *VviMYBPA1* expression was also found to be upregulated with high temperatures [37], which was in agreement with our study. *VviMYBC2-L1* was a negative repressor of the proanthocyanidin biosynthesis but showed opposite trends in the two varieties in terms of the winter vs. the summer season. The regulator of *VviMYBF1* was involved in the regulation of flavonol biosynthesis, which could induce the expression of flavonol synthase (VviFLS1/VviFLS4), a key step of the initial flavonol pathway [38]. However, *VviMYBF1* was downregulated in winter berries in both of ‘Muscat Hamburg’ and ‘Victoria’ grapes, which showed opposite trends to the upregulated *VviFLS4* and higher flavonol concentration in the winter season berries. So there might be other transcription factors that play an important role in regulating *VviFLS4*. For example, *VvibZIPC22* expression was induced by ultraviolet light (UV), accompanied by the expression of *VviFLS4* and the accumulation of flavonols [39]. In the winter season berries, the expression of *VvibZIPC22* was higher than that in the summer berries at E-L 35 and E-L 36, which might lead to a higher *VviFLS4* expression.

## 4. Discussion

### 4.1. Effects of Growing Season on Anthocyanin Composition of ‘Muscat Hamburg’ Grape

Under the double cropping system, the anthocyanin concentration in the winter season berries was at least seven-fold higher than that in the summer season berries in our study. The same result was also found by previous studies [6,7]. Xu et al. [7] showed that the total anthocyanin content in ‘Kyoho’ skins in the winter season berries was about five-fold higher than the value of the corresponding summer season berries. Zhu et al. [6] reported that anthocyanin content in the winter season berries of ‘Muscat Hamburg’ could be eleven-fold higher than that in the summer season season. The vast variation between the summer and the winter season berries in the anthocyanin accumulation showed a great range of phenotypic plasticity caused by climate factors. Among all climate factors, the high-temperature effect was the most conspicuous in our study. However, a different result was found by Chou and Li [3] that anthocyanin concentration in the ‘Kyoho’ grape was not affected by the seasonal variations between the summer and winter cropping system. They inferred that the complex environmental or physiological factors might overwhelm the influence of the temperature on the anthocyanin accumulation, although the temperature of the post veraison period was also higher in the summer cropping cycle than in the winter cropping cycle in their study [3]. Furthermore, the temperature variation between the seasons in their research was not notable, while in our study, the average daily temperature in the summer season could be 9 °C higher than that in the winter season, which should be resulted from the different climates between the studies. The expressions of *VviUFGT* and *VviAOMT* were upregulated in the winter season berries, which was consistent with the coordinated expression of the upstream genes in our study. In previous studies, the expression of the *UFGT* gene was significantly down-regulated by the high temperature until the mid-ripening in the ‘Malbec’ grapes [40] and from the mid-ripening to maturity in the ‘Sangiovese’ grapes [41]. In the winter season, almost no high-temperature weather occurred, which provided beneficial conditions for the anthocyanin biosynthesis.

Among all climate factors, the temperature should also be the dominant one in affecting the proportion of each group of anthocyanins. In a previous study, Tarara et al. [42] found that higher berry temperatures led to a higher proportion of the malvidin-based anthocyanins while decreasing the cyanidin-based anthocyanins, which was in agreement with our study. Acylated anthocyanins were known to be more stable than their nonacylated counterparts [43]. In the summer season, berries suffered more high-temperature stress than those in the winter, leading to the degradation of various anthocyanins types. Among all anthocyanins, non-acylated and non-methylated anthocyanins were easily degraded, leading to a higher proportion of the acylated and the methylated anthocyanins. The same result was also found by Tarara et al. [42]. They showed that grape berries might shunt more of the available anthocyanin toward acylation in response to the temperature stress, with the potential advantage to the plant of color stability within the vacuole because of the stability of the acylated compounds. As for the increased proportion of methylated and 3′5′-hydroxylated anthocyanins in the summer berries, the increased proportion of malvidin-based anthocyanins was the cause.

### 4.2. Effects of Growing Season on Berries Flavonol Composition

Flavonol was well known to be positively correlated with sunlight exposure. The biosynthesis of flavonol was upregulated by the solar radiation, leaving a fingerprint on the flavonol profile [44]. However, in our study, although the 2015 summer season had the most abundant sunshine hours and cumulative PAR, the flavonol concentration of the summer season berries was lower than that of the winter season berries in the ‘Muscat Hamburg’ grapes. The same results were also found by Chen et al. [1] and Zhu et al. [6] that the flavonol concentration in the winter season berries was higher than those in the summer season berries. Although flavonols responses to the temperature seemed to vary depending on the experimental parameters, the studies with whole-vine and the detached berry levels reported an effect on the flavonol concentration [41,45,46]. Pastore et al. [41] reported that in a low temperature condition, the flavonol concentration in berries was three times of that in the high-temperature conditions. In the summer season, the whole vine suffered more heat stress, thus causing a general decrease in metabolism at the whole vine level. The reduced primary metabolites as flavonoid precursors could impact the downstream production of flavonols [45]. So the decrease of the flavonol concentration in the summer season berries might mainly result from the high-temperature stress, which had a greater effect than the offset from the higher light radiation in the summer season of a certain vintage. However, compared to the summer (2014 summer vs. 2015 summer) or the winter (2014 winter vs. 2015 winter) growing seasons, more sunshine hours during the growing period were associated with the higher flavonol concentration in the berries in our study. Compared to the summer season berries, the winter season berries had higher expressions of *VviFLS4* and *VviFLS5* than the summer season berries in both of the two varieties. In general, the white grapes seemed to have a lower flavonol concentration than the red grapes. Mattivi et al. [23] showed that the total amount of flavonols found after the hydrolysis of the grape extracts ranged from 3.81 to 80.37 mg/kg, with a mean of 32.46 mg/kg in the 64 tested red varieties and from 1.36 to 30.21 mg/kg, with a mean of 10.83 mg/kg in the 36 tested white varieties. However, in our study, in the 2014 winter season berries of ‘Victoria’ grapes, the total flavonol concentration could be up to 100 mg/kg FW, which might owe to the high expression of *VviFLS4* in the berries.

### 4.3. Effects of Growing Season on Berries Flavanol Composition

The influence of growing seasons on flavanols seemed to be variety dependent, which was different from the consistent influence found in the anthocyanins and flavonols accumulation in our study. Similarly, in a previous study, Zhu et al. [6] reported that the winter season berries of ‘Khoyo’ and ‘Muscat Hamburg’ had higher flavanol concentrations than the summer season berries at harvest, while no significant difference was found in the summer and the winter season berries of the ‘NW196′ grape. However, Xu et al. [7] showed that flavanols in the skin of the winter season berries were higher than those of the summer season berries for all of the cultivars they investigated. Although flavanols shared the same common upstream steps with flavonols and anthocyanins, the high temperature seemed to have a minor influence on the flavanol concentration in our study. Until now, the effect of temperature on the flavan-3-ol biosynthesis and PA accumulation was not well understood [45]. Some studies reported that no effect on the skin PAs when the high temperature treatments were applied at the whole-vine level [41,47], while other studies also reported a decrease in the skin PAs was correlated with higher temperature [48,49]. The formation of flavanols in skins mostly occurred in the early stage of the berry development, starting from the fruit-set with maximum levels observed before veraison [50]. Under the double cropping system, the summer season had less heat pressure and the winter season had a warm condition in the former stage of the berry development, which might cause little variation in the flavanol concentration between the summer and the winter season berries. *VviLAR1* (VIT_201s0011g02960) and *VviLAR2* (VIT_217s0000g04150) were upregulated in the ‘Muscat Hamburg’ winter season berries, which led to a higher flavanol concentration than the summer season berries in 2014. However, in the ‘Victoria’ grapes, *VviLAR2* was downregulated in the winter season berries, which was in agreement with the higher flavanol concentration found in the 2014 summer season berries than those in the winter season berries in the previous analysis.

## 5. Conclusions

In this study, the cool weather conditions in the winter season made the grape berries accumulate more phenolic compounds than those in the summer season under the double cropping system. Although this cropping system could avoid intense rainfall and heatwave as much as possible, the high summer season temperature still inhibited the flavonoid metabolism to a great extent. For anthocyanins, the non-acylated and non-methylated anthocyanins were easily degraded in response to the high temperature, thus leading to the higher acylated and methylated anthocyanin proportions in the summer season berries. Flavanols were more stable to the climate changes than anthocyanins and flavonols, because the flavanol concentration did not show a significant decrease in the summer season berries. Most of the genes and transcription factors related to the flavonoid biosynthesis were upregulated in the winter season berries, which was consistent with the results found in the metabolites. The variation in *VviLARs* expression between ‘Muscat Hamburg’ and ‘Victoria’ grapes might be the reason why flavanols showed different trends in response to the climate variation among seasons. The extreme weather conditions in the summer season provide possible insights into how global warming or climate changes would impact viticulture in the future.

## Figures and Tables

**Table 1:** Meteorological data in the growing seasons of ‘Muscat Hamburg’ and ‘Victoria’ grapes in 2014 and 2015.

Year	Season	Variety	Development Stages	GDD (°C)	Average Daily Temperature (°C)	High Temperature (>35 °C) Hours	Cumulative PAR (10^3^ μmol/m^2^/s)	Cumulative Sunshine Hours (h)	Cumulative Rainfall (mm)
2014	Summer	MH and V	Stage I	1021.4	27.9	209	23.7	204.7	147.4
			Stage II	358.7	29.9	89	7.2	68.5	60.8
			Stage III	146.3	30.9	39	3.0	24.6	5.7
			Whole season	1526.4	28.6	337	33.9	297.8	213.9
	Winter	MH and V	Stage I	958.1	23.1	89	27.3	341.1	447.1
			Stage II	85.9	13.4	0	6.6	86.1	30.3
			Stage III	54.8	13.9	0	4.5	68.8	16.8
			Whole season	1098.8	19.3	89	38.4	506.3	494.2
2015	Summer	V	Stage I	968.9	28.9	205	27.5	276.5	306.8
			Stage II	443.1	31.1	96	11.0	99.8	66.2
			Stage III	262.7	31.9	61	6.8	78.8	16.0
			Whole season	1674.7	29.7	362	45.2	455.1	386.0
	Summer	MH	Stage I	1085.8	29.0	229	30.1	295.1	346.4
			Stage II	771.0	31.4	229	20.8	224.4	39.6
			Stage III	238.8	28.4	37	5.65	45.6	213.8
			Whole season	2095.6	29.7	495	56.4	565.1	599.8
	Winter	MH and V	Stage I	854.0	25.3	54	24.0	264.8	143.6
			Stage II	236.1	21.2	1	57.8	40.9	59.4
			Stage III	256.9	15.1	0	94.7	73.4	173.0
			Whole season	1347.0	20.3	55	39.2	379.1	376.0

GDD, growing degree days (based on 10 °C). PAR, photosynthetically active radiation. Stage I was from full bloom to veraison beginning; Stage II was from veraison beginning to veraison completement; Stage III was from veraison completement to harvest.

**Table 2:** Anthocyanin concentration (mg/kg FW) in ‘Muscat Hamburg’ grapes in 2014 and 2015 under the double cropping system.

Compound	2014 Summer	2014 Winter	2015 Summer	2015 Winter	S	V	S × V
Delphinidin-3-*O*-glucoside	2.1 ± 0.5c	31.6 ± 5.0a	1.0 ± 0.1d	13.1 ± 3.6b	***	***	**
Cyanidin-3-*O*-glucoside	5.1 ± 1.0c	159.4 ± 20.0a	1.5 ± 0.4d	35.6 ± 0.8b	***	***	***
Petunidin-3-*O*-glucoside	2.4 ± 0.6c	31.3 ± 5.5a	1.1 ± 0.2c	10.8 ± 2.3b	***	***	***
Peonidin-3-*O*-glucoside	48.4 ± 6.2c	395.5 ± 16.9a	12.0 ± 2.1d	223.5 ± 21.2b	***	***	***
Malvidin-3-*O*-glucoside	25.2 ± 4.6b	104.4 ± 6.7a	10.4 ± 1.1c	89.2 ± 8.6a	***	**	ns
Delphinidin-3-*O*-acetylglucoside	0.2 ± 0.0b	0.7 ± 0.1a	0.3 ± 0.1b	0.3 ± 0.0b	**	ns	**
Peonidin-3-*O*-acetylglucoside	0.6 ± 0.1b	1.3 ± 0.1a	0.5 ± 0.1b	1.0 ± 0.1a	***	*	ns
Malvidin-3-*O*-acetylglucoside	0.4 ± 0.1b	0.5 ± 0.1ab	0.7 ± 0.1a	0.7 ± 0.1a	ns	**	ns
Cyanidin-3-*O*-coumarylglucoside	0.6 ± 0.1bc	1.6 ± 0.2a	0.3 ± 0.1c	0.9 ± 0.1b	***	***	**
Petunidin-3-*O*-coumarylglucoside	0.2 ± 0.0b	0.5 ± 0.1a	0.3 ± 0.0b	0.3 ± 0.1b	**	ns	**
Peonidin-3-*O*-coumarylglucoside	4.5 ± 0.6b	9.8 ± 1.0a	1.5 ± 0.2b	5.3 ± 0.3a	***	ns	*
Malvidin-3-*O*-coumarylglucoside	2.4 ± 0.2c	3.6 ± 0.1b	4.1 ± 0.2b	7.6 ± 0.6a	***	***	***
ΣDp (%)	2.5 ± 0.2b	4.3 ± 0.5a	3.5 ± 0.2ab	3.4 ± 0.8ab	*	ns	**
ΣPt (%)	2.8 ± 0.3b	4.3 ± 0.6a	3.9 ± 0.0ab	2.9 ± 0.8b	ns	ns	**
ΣMv (%)	30.3 ± 0.8b	14.7 ± 0.6d	43.5 ± 2.0a	24.8 ± 0.9c	***	***	ns
ΣCy (%)	6.2 ± 0.4c	21.7 ± 1.4a	5.1 ± 0.5c	9.3 ± 0.6b	***	***	***
ΣPn (%)	58.3 ± 1.6a	55.0 ± 2.6b	44.0 ± 1.3c	59.7 ± 1.0a	***	**	***
Total anthocyanin concentration	92.0 ± 13.8c	740.9 ± 47.4a	35.1 ± 4.8d	394.0 ± 30.4b	***	***	***

Values are reported as means ± SD of three biological replicates. Different letters within a row indicate significant differences among treatments (Duncan’s multiple range test at *p* < 0.05). In two-way ANOVA, “S” indicates season effect, “V” indicates vintage effect, “S × V” indicates interaction effect of season and vintage, “*” indicates 0.05 > *p* ≥ 0.01, “**” indicates 0.01 > *p* ≥ 0.001, “***” indicates 0.001 > *p*, “ns” indicates *p* > 0.05.

**Table 3:** Flavonol concentration (mg/kg FW) in ‘Muscat Hamburg’ and ‘Victoria’ grapes in 2014 and 2015 under the double cropping system.

Compound	‘Muscat Hamburg’				‘Victoria’			
	2014 Summer	2014 Winter	2015 Summer	2015 Winter	2014 Summer	2014 Winter	2015 Summer	2015 Winter
Myglu	0.3 ± 0.2c	3.5 ± 0.5a	0.3 ± 0.0c	0.8 ± 0.1b	-	-	-	-
Mygal	0.1 ± 0.1c	0.6 ± 0.1a	0.4 ± 0.0b	0.7 ± 0.1a	-	-	-	-
Mygluc	2.7 ± 0.5c	20.7 ± 1.7a	2.8 ± 0.3c	14.4 ± 2.8b	-	-	-	-
Qugal	0.2 ± 0.0d	1.6 ± 0.1a	0.5 ± 0.1c	0.7 ± 0.1b	0.1 ± 0.0c	6.9 ± 0.9a	0.9 ± 0.4bc	1.6 ± 0.2b
Qugluc	2.6 ± 0.2d	10.1 ± 0.3a	4.0 ± 0.3c	6.7 ± 0.8b	5.8 ± 0.9c	22.3 ± 1.6a	15.2 ± 2.2b	15.9 ± 2.1b
Qurut	-	-	-	-	0.5 ± 0.1c	4.5 ± 1.0a	1.1 ± 0.2bc	2.1 ± 0.2b
Quglu	2.5 ± 0.4d	20.2 ± 1.2a	7.2 ± 1.0c	10.9 ± 1.2b	1.3 ± 0.4c	38.0 ± 4.5a	9.0 ± 3.4b	10.6 ± 1.0b
Laglu	0.2 ± 0.0b	0.54 ± 0.1 a	0.58 ± 0.1a	0.67 ± 0.2a	-	-	-	-
Kagal	Trace	0.2 ± 0.1	Trace	Trace	Trace	7.1 ± 0.8a	0.5 ± 0.2c	1.5 ± 0.1b
Kagluc	0.1 ± 0.0c	1.0 ± 0.3a	0.3 ± 0.0c	0.5 ± 0.1b	0.1 ± 0.0d	1.0 ± 0.1a	0.6 ± 0.2b	0.4 ± 0.0c
Kaglu	Trace	0.7 ± 0.1a	0.1 ± 0.0c	0.4 ± 0.1b	0.10 ± 0.1d	26.7 ± 2.8a	1.7 ± 0.8c	5.9 ± 0.6b
Isglu	0.6 ± 0.1c	2.4 ± 0.2a	1.1 ± 0.1b	2.1 ± 0.3a	Trace	0.4 ± 0.0a	Trace	0.1 ± 0.0b
Syglu	0.1 ± 0.0c	0.2 ± 0.1c	0.3 ± 0.0b	0.4 ± 0.1a	-	-	-	-
ΣMy (%)	32.7 ± 1.6b	40.3 ± 1.0a	20.1 ± 1.8c	41.4 ± 4.3a	-	-	-	-
ΣQu (%)	56.4 ± 1.7b	51.7 ± 1.0c	66.4 ± 1.8a	48.0 ± 4.0c	97.6 ± 0.6a	67.1 ± 0.3d	90.7 ± 1.9b	79.4 ± 0.5c
ΣKa (%)	2.5 ± 0.1b	0.9 ± 0.1d	3.3 ± 0.3a	1.8 ± 0.1c	2.4 ± 0.6d	32.6 ± 0.3a	9.3 ± 1.9c	20.3 ± 0.5b
ΣLa (%)	1.2 ± 0.1b	2.9 ± 0.7a	2.6 ± 0.0a	2.3 ± 0.1a	-	-	-	-
ΣIs (%)	5.9 ± 0.3ab	3.9 ± 0.1c	6.2 ± 0.2a	5.5 ± 0.3b	-	0.3 ± 0.0a	0.1 ± 0.0b	0.3 ± 0.0a
ΣSy (%)	1.3 ± 0.2ab	0.3 ± 0.1c	1.5 ± 0.2a	1.0 ± 0.0b	-	-	-	-

Myglu, Myricetin-3-*O*-glucoside; Mygal, myricetin-3-*O*-galactoside; Mygluc, myricetin-3-*O*-glucuronide; Qugal, quercetin-3-*O*-galactoside; Qugluc, quercetin-3-*O*-glucuronide; Qurut, quercetin-3-*O*-rutinoside; Quglu, quercetin-3-*O*-glucoside; Laglu, laricitrin-3-*O*-glucoside; Kagal, kaempferol-3-*O*-galactoside; Kagluc, kaempferol-3-*O*-glucuronide; Isglu, isorhamnetin-3-*O*-glucoside; Syglu, syringetin-3-*O*-glucoside. Values are reported as means ± SD of three biological replicates. Different letters within the same variety indicate significant differences among seasons (Duncan’s multiple range test at *p* < 0.05). -, not detected.

**Table 4:** Flavanol concentration (mg/kg FW) in ‘Muscat Hamburg’ and ‘Victoria’ grapes in 2014 and 2015 under the double cropping system.

Compound	‘Muscat Hamburg’				‘Victoria’			
	2014 Summer	2014 Winter	2015 Summer	2015 Winter	2014 Summer	2014 Winter	2015 Summer	2015 Winter
(+)–Catechin	83.7 ± 26.8a	99.5 ± 7.2a	78.4 ± 7.6a	90.3 ± 6.7a	57.4 ± 9.0a	42.3 ± 15.0ab	25.3 ± 8.9b	31.1 ± 9.0b
(–)–Epicatechin	457.5 ± 11.3c	605.7 ± 10.9a	474.5 ±38.6bc	509.9 ± 23.3b	387.1 ± 23.6a	316.1 ± 60.1b	220.0 ± 2.6c	244.7 ± 48.8c
(–)–Epicatechin-3-*O*-gallate	68.0 ± 11.3a	35.8 ± 11.4b	39.0 ± 3.6b	64.3 ± 7.9a	77.5 ± 4.6a	42.2 ± 17.8b	15.3 ± 5.0c	32.7 ± 6.2b
(–)–Epigallocatechin	25.1 ± 5.8c	34.0 ± 11.8bc	79.2 ± 2.4a	59.1 ± 27.3ab	14.5 ± 5.0c	16.2 ± 12.7bc	29.0 ± 1.3ab	34.6 ± 8.8a
Terminal subunits	71.5 ± 21.6a	81.0 ± 8.1a	77.5 ± 7.7a	81.8 ± 6.8a	41.4 ± 9.1a	27.0 ± 14.4ab	19.6 ± 1.7b	24.5 ± 8.9ab
Extension subunits	555.6 ± 118.4a	681.0 ± 12.4a	585.0 ± 40.5a	627.7 ± 27.7a	491.0 ± 20.9a	383.3 ± 83.1b	268.6 ± 5.9c	313.8 ± 63.3bc
Free monomers	7.1 ± 1.0c	12.9 ± 2.1a	8.6 ± 0.1b	14.1 ± 1.4a	4.1 ± 2.5ab	6.5 ± 1.4a	2.3 ± 0.5b	4.8 ± 0.3ab

Values are reported as means ± SD of three biological replicates. Different letters within a row indicate significant differences among treatments (Duncan’s multiple range test at *p* < 0.05).

## Data Availability

The data showed in this study are contained within the article.

## Supplementary Materials

The following are available online at https://www.mdpi.com/article/10.3390/foods11010048/s1, Table S1: Grapevine growth stages of ‘Muscat Hamburg’ and ‘Victoria’ grapes under the double cropping system. Table S2: Expression profiles of the genes related to the flavonoid biosynthesis pathway in ‘Muscat Hamburg’ and ‘Victoria’ grapes in the year of 2014. Figure S1: PCA (**a**, score plot, the samples were marked according to the development stages; **b**, score plot, the samples were marked according to different seasons; **c**, loading plot) based on the concentration of individual phenolic compounds in ‘Muscat Hamburg’ grapes and ‘Victoria’ grapes in the years of 2014 and 2015 under the double cropping system. Figure S2: The 200 permutation tests were based on the OPLS-DA model for discriminating summer and winter berries.
