# Supplementary material for: Effect of the Seasonal Climatic Variations on the Flavonoid Accumulation in Vitis vinifera cvs. ‘Muscat Hamburg’ and ‘Victoria’ Grapes under the Double Cropping System
Source: Foods. 2021 Dec 25;11(1):48. doi: 10.3390/foods11010048 (PMC8750161; doi:10.3390/foods11010048)
Supplement: Supplementary file 1 [file foods-11-00048-s001.zip › Supplementary Figures.pdf]

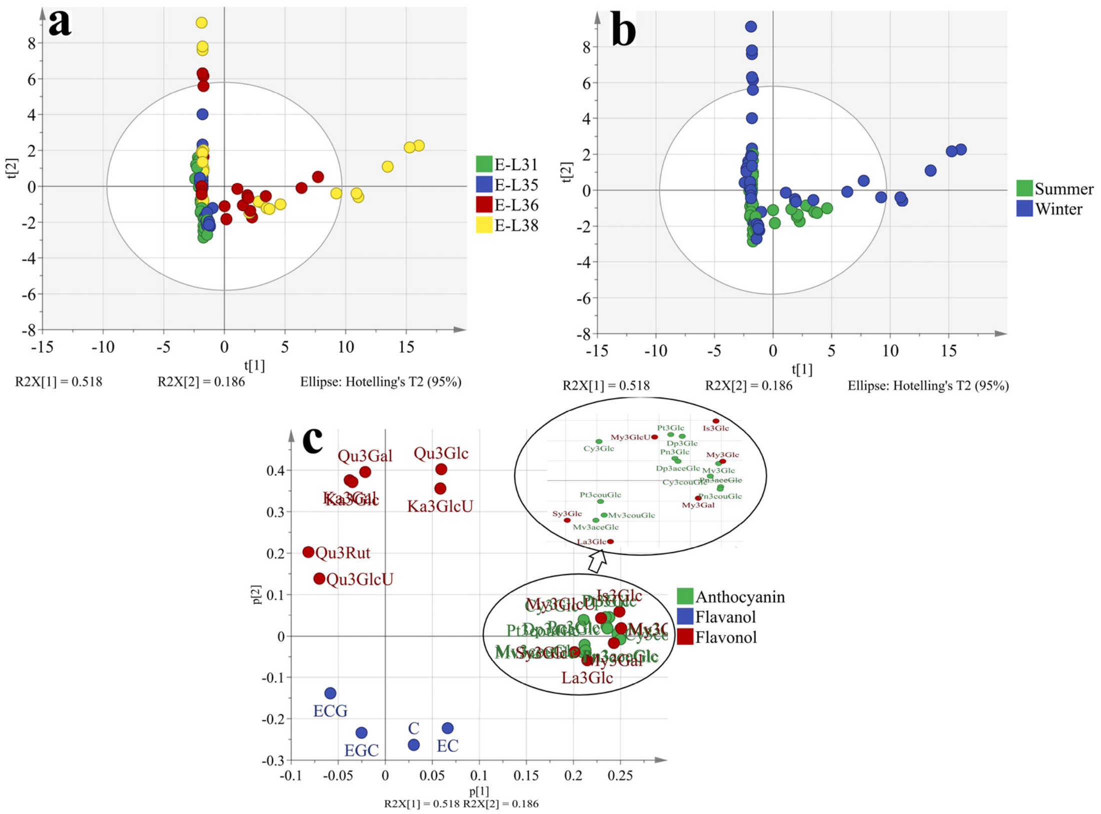

Supplementary Figure S1. PCA (**a**, score plot, the samples were marked according to the development stages; **b**, score plot, the samples were marked according to different seasons; c, loading plot) based on the concentration of individual phenolic compounds in 'Muscat Hamburg' grapes and 'Victoria' grapes in the years of 2014 and 2015 under the double cropping system. Dp3Glc, delphinidin-3-*O*-glucoside; Cy3Glc, cyanidin-3-*O*-glucoside; Pt3Glc, petunidin-3-*O*-glucoside; Pn3Glc, peonidin-3-*O*-glucoside; Mv3Glc, malvidin-3-*O*-glucoside, Dp3aceGlc, Delphinidin-3-*O*-acetyl-glucoside; Pn3aceGlc, peonidin-3-*O*-acetylglucoside; Mv3aceGlc, malvidin-3-*O*-acetyl-glucoside; Cy3couGlc, cyanidin-3-*O*-coumaryl-glucoside; Pt3couGlc, petunidin-3-*O*-coumaryl-glucoside; Pn3couGlc, peonidin-3-*O*-coumaryl-glucoside; Mv3couGlc, malvidin-3-O-coumarylglucoside; My3GlcU, myricetin-3-*O*-glucuronide; My3Gal, myricetin-3-*O*-galactoside; My3Glc, myricetin-3-*O*-glucoside; Qu3Gal, quercetin-3-*O*-galactoside; Qu3GlcU, quercetin-3-*O*-glucuronide; Qu3Rut, quercetin-3-*O*-rutinoside; Qu3Glc, quercetin-3-*O*-glucoside; La3Glc, laricitrin-3-*O*-glucoside; Ka3Gal, kaempferol-3-*O*-galactoside; Ka3GlcU, kaempferol-3-*O*-glucuronide; Ka3Glc, kaempferol-3-*O*-glucoside; Is3Glc, isorhamnetin-3-*O*-glucoside; Sy3Glc, syringetin-3-*O*-glucoside; ECG, epicatechin-3-*O*-gallate; EGC, epigallocatechin; C, catechin; EC, Epicatechin

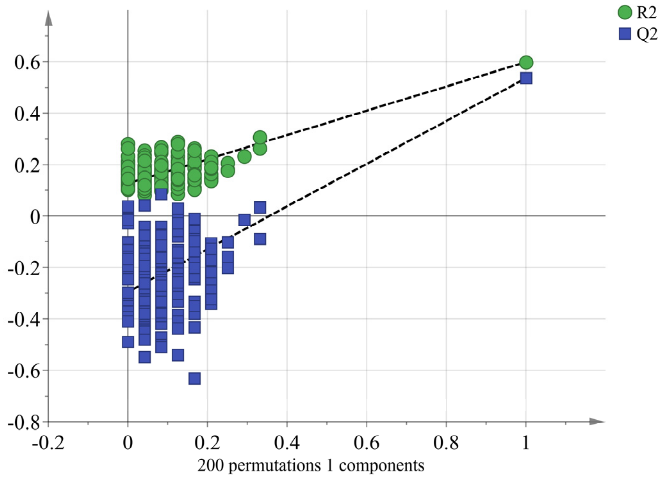

Supplementary Figure S2. The 200 permutation tests were based on the OPLS-DA model for discriminating summer and winter berries.
